# Supplementary material for: A Critical View of the Application of the APEX Software (Aqueous Photochemistry of Environmentally-Occurring Xenobiotics) to Predict Photoreaction Kinetics in Surface Freshwaters
Source: Molecules. 2019 Dec 18;25(1):9. doi: 10.3390/molecules25010009 (PMC7017383; doi:10.3390/molecules25010009)
Supplement: Supplementary file 1 [file molecules-25-00009-s001.zip › 3.2.4_gcc-4.4.0.zip!/3.2.4_gcc-4.4.0/3.2.4_gcc-4.4.0/doc/octave/html/interpreter/Evaluation-Notation.html]

Evaluation Notation - Untitled


Next: Printing Notation,
Previous: Fonts,
Up: Conventions


---

#### 1.3.2 Evaluation Notation

In the examples in this manual, results from expressions that you
evaluate are indicated with =>. For example,

```
     sqrt (2)
          => 1.4142
```

You can read this as “`sqrt (2)` evaluates to 1.4142”.

In some cases, matrix values that are returned by expressions are
displayed like this

```
     [1, 2; 3, 4] == [1, 3; 2, 4]
          => [ 1, 0; 0, 1 ]
```

and in other cases, they are displayed like this

```
     eye (3)
          =>  1  0  0
              0  1  0
              0  0  1
```

in order to clearly show the structure of the result.

Sometimes to help describe one expression, another expression is
shown that produces identical results. The exact equivalence of
expressions is indicated with ==. For example,

```
     rot90 ([1, 2; 3, 4], -1)
     ==
     rot90 ([1, 2; 3, 4], 3)
     ==
     rot90 ([1, 2; 3, 4], 7)
```
